# m^6^A Reader: Epitranscriptome Target Prediction and Functional Characterization of *N*^6^-Methyladenosine (m^6^A) Readers

**DOI:** 10.3389/fcell.2020.00741

**Published:** 2020-08-11

**Authors:** Di Zhen, Yuxuan Wu, Yuxin Zhang, Kunqi Chen, Bowen Song, Haiqi Xu, Yujiao Tang, Zhen Wei, Jia Meng

**Affiliations:** ^1^Department of Biological Sciences, Xi’an Jiaotong-Liverpool University, Suzhou, China; ^2^Institute of Ageing and Chronic Disease, University of Liverpool, Liverpool, United Kingdom; ^3^Department of Mathematical Sciences, Xi’an Jiaotong-Liverpool University, Suzhou, China; ^4^Institute of Integrative Biology, University of Liverpool, Liverpool, United Kingdom; ^5^AI University Research Centre, Xi’an Jiaotong-Liverpool University, Suzhou, China

**Keywords:** *N6*-methyladenosine, m^6^A reader, machine learning (ML), YTH domain, eIF3a

## Abstract

*N*^6^-methyladenosine (m^6^A) is the most abundant post-transcriptional modification in mRNA, and regulates critical biological functions via m^6^A reader proteins that bind to m^6^A-containing transcripts. There exist multiple m^6^A reader proteins in the human genome, but their respective binding specificity and functional relevance under different biological contexts are not yet fully understood due to the limitation of experimental approaches. An *in silico* study was devised to unveil the target specificity and regulatory functions of different m^6^A readers. We established a support vector machine-based computational framework to predict the epitranscriptome-wide targets of six m^6^A reader proteins (YTHDF1-3, YTHDC1-2, and EIF3A) based on 58 genomic features as well as the conventional sequence-derived features. Our model achieved an average AUC of 0.981 and 0.893 under the full-transcript and mature mRNA model, respectively, marking a substantial improvement in accuracy compared to the sequence encoding schemes tested. Additionally, the distinct biological characteristics of each individual m^6^A reader were explored via the distribution, conservation, Gene Ontology enrichment, cellular components and molecular functions of their target m^6^A sites. A web server was constructed for predicting the putative binding readers of m^6^A sites to serve the research community, and is freely accessible at: http://m6areader.rnamd.com.

## Introduction

In the exploration of RNA epigenetics, more than 150 types of RNA modification have been identified (Boccaletto et al., 2018). The methylation of adenosine at the N6 position (m^6^A) is the most prevalent post-transcriptional modification in the mRNA (Meyer and Jaffrey, 2017), which was discovered in a wide range of eukaryotic RNAs (Adams and Cory, 1975) as well as viral RNAs (Gokhale et al., 2016). m^6^A was considered as a potential mRNA processing regulator in 1970s (Desrosiers et al., 1974), and subsequent studies noticed intensive functions of it (Patil et al., 2018), including cardiac gene expression (Kmietczyk et al., 2019), cell growth, neuronal development (Chen J. et al., 2019), stress response (Engel et al., 2018), translation initiation, and stabilizing junctional RNA (Liu B. et al., 2018).

Similar to other epigenetic modifications, m^6^A is thought to be dynamic and reversible (Song et al., 2019). It can be installed by methyltransferase (writers) or removed by demethylase (erasers). This internal modification also attracts specific binding proteins, namely readers, which bind selectively to m^6^A-containing transcripts (Liao et al., 2018). Additionally, m^6^A performs many functions through interacting with “reader” proteins (Hazra et al., 2019). The most widely studied readers are YT521-B homology (YTH) family of proteins, which possess the evolutionarily conserved YTH domain that recognizes m^6^A mark. The YTH domain consists of 100–150 residues and adopts alpha/beta fold, with 4–5 alpha helices surrounding a curved six-stranded beta sheet (Zhang et al., 2010). In human, five m^6^A readers were reported to have the YTH domain, namely YTHDF1,2,3 and YTHDC1,2. However, the YTH domain is not indispensable for m^6^A readers, a subunit of translation initiation complex factor EIF3 complex, called EIF3A, was reported as an m^6^A reader lacking YTH domain (Meyer et al., 2015).

The m^6^A reader YTHDC1 is predominantly found in the nucleus, while YTHDC2 and YTHDF1,2,3 are cytoplasmic (Patil et al., 2016). YTHDC1 and YTHDC2 are unrelated to other members of the YTH family based on amino acid sequence, size or overall YTH domain organization (Patil et al., 2018). By contrast, YTHDF family comprises three paralogs, YTHDF1-3, that share high sequence identity with about 85% of sequence similarity (Hazra et al., 2019). YTHDC1 and three YTHDF proteins contain a single C-terminal YTH domain that binds to m^6^A marker by a segment rich of proline, glutamate and aspartate. Compared to other YTH domain-containing proteins, whose YTH domains are embedded in low complexity regions, YTHDC2 has a unique multidomain structure (Hazra et al., 2019). N-terminal R3H domain, central DEAH-box helicase domain and helicase associated 2 domain are also found in YTHDC2 apart from the C-terminal YTH domain. Different from the structures of five YTH domain-containing proteins, EIF3 is a large multiprotein complex comprising 13 subunits (Meyer et al., 2015). The EIF3 binding sites are predominantly mapped at the 5′ untranslated region (5′ UTR) (Lee et al., 2015), whereas the binding sites of YTH domain-containing proteins are usually located near the stop codon.

In addition to different cellular locations and structures, m^6^A readers appear to function through various post-transcriptional control mechanisms to regulate RNAs dynamically. Human YTHDC1 has been demonstrated to participate in RNA splicing by interacting with serine/arginine splicing factor SRSF3, which is involved in exon inclusion and exclusion splicing (Ye et al., 2017). As a putative RNA helicase, YTHDC2 enhances the translation of target RNAs and reduces the abundance of target RNAs (Hsu et al., 2017). YTHDF2 is verified to decrease the stability and control the lifetime of its targeted methylated mRNA transcripts (Du et al., 2016), while YTHDF1 ensures efficient protein expression from their shared regions (Wang et al., 2015). YTHDF3, the third member of YTHDF family, has been proposed to share common targets (about 60%) with both YTHDF1 and YTHDF2 (Shi et al., 2017). This suggests potential coordination in regulating gene expression by YTHDF family proteins. YTHDF3 can promote the function of YTHDF1 by interacting with some ribosomal proteins to facilitate mRNA translation. When associating with YTHDF2, YTHDF3 could participate in mRNA decay. In addition to the five members of YTH family, EIF3A plays an important role in biological processes as well. It can act as both repressor and activator of cap-dependent transcript-specific translation through directly binding to m^6^A marked mRNA sequence (Lee et al., 2015).

Since the five YTH family proteins (YTHDC1-2 and YTHDF1-3) and EIF3A present distinctive structures and properties, it is worth studying the preferential binding sites in the m^6^A marked transcripts for each m^6^A reader.

Single base resolution techniques such as miCLIP (Linder et al., 2015) are developed and are fairly effective on screening m^6^A sites, and it is usually based on the iCLIP or Par-CLIP approach (Meyer et al., 2015) to identify the binding sites of each m^6^A reader. As these wet-lab experiments are costly and laborious, computational methods may provide a viable avenue. To date, a large number of RNA methylation sites have been reported, providing sufficient information for effective computational prediction. A huge amount of data extracted from experiments encouraged the establishment of a number of effective m^6^A site predictors, including WHISTLE (Chen K. et al., 2019), SRAMP (Zhou et al., 2016), BERMP (Huang et al., 2018), and Gene2vec (Zou et al., 2019). However, to our knowledge, the prediction dedicated to the target specificity of the readers is absent. In this project, we constructed a predictor, m^6^A reader, to distinguish the substrate of each m^6^A reader. A comprehensive analysis of these readers was then performed, including the analysis of distribution, conservation, GO enrichment, cellular components and molecular functions of their respective epitranscriptome target sites.

## Materials and Methods

### Collection of m^6^A Sites and the Target Sites of m^6^A Readers

The transcriptome-wide m^6^A sites were collected from 17 different conditions generated from 6 different epitranscriptome profiling approaches of base-resolution or high resolution (Table 1).

**TABLE 1:** Base-resolution or high resolution datasets of m^6^A sites.

Dataset	Technique	Cell line	GEO	References
S1	miCLIP	MOLM13	GSE98623	Vu et al., 2017
S2		HEK293	GSE63753	Linder et al., 2015
S3		HepG2	GSE73405	Meyer et al., 2015
S4		HEK293T	GSE122948	Boulias et al., 2019
S5		HepG2	GSE121942	Huang et al., 2019
S6		HCT116	GSE128699	van Tran et al., 2019
S7	m^6^A-CLIP	HeLa	GSE86336	Ke et al., 2017
S8		CD8T	GSE71154	Ke et al., 2015
S9		A549		
S10	MAZTER-seq	HEK293T	GSE122961	Garcia-Campos et al., 2019
S11		ESC		
S12	m^6^A-REF-seq	HEK293	GSE125240	Zhang et al., 2019c
S13		Brain		
S14		Kidney		
S15		Liver		
S16	PA-m^6^A-seq	HeLa	GSE54921	Chen K. et al., 2015a
S17	m^6^A-seq (improved protocol)	A549	GSE54365	Schwartz et al., 2014

In this study, we consider the binding sites of six m^6^A readers identified by Par-CLIP or iCLIP approaches. Specifically, a total of 16,664 m^6^A sites located on 4,722 different genes reported by four experiments were considered as the target sites of YTHDC1, and 1,234 sites on 275 genes identified by two experiments were considered as the target sites for YTHDC2. For the three proteins from YTHDF family, three experiments for each reader proposed 25,597, 28,970, and 7,253 target sites located on 6,714, 6,677, and 3,495 genes for YTHDF1, YTHDF2, and YTHDF3, respectively. Two CLIP experiments conducted on HEK2937T cell line discovered 756 sites located in 470 genes on marked RNA transcripts, which are targeted by EIF3A. The testing datasets and training datasets are strictly segregated under all conditions. Detailed information of the target sites of m^6^A readers analyzed in this study was summarized in Table 2.

**TABLE 2:** Target sites of m^6^A readers identified by Par-CLIP or iCLIP.

Dataset	Reader	Source	Site #	Total #	Gene #	Cell line
D1	YTHDC1	GSE74397 (Roundtree et al., 2017)	482	16,664	4,722	HeLa
D2		GSE58352 (Xu et al., 2014)	2,633			
D3		GSE71096 (Xiao et al., 2016)	2,430			
D4		GSE78030 (Patil et al., 2016)	12,309			HEK293T
D5	YTHDC2	GSE98085 (Hsu et al., 2017)	1,183	1,234	275	HeLa
D6		GSE78030 (Patil et al., 2016)	131			HEK293T
D7	YTHDF1	GSE63591 (Wang et al., 2015)	4,541	25,597	6,714	HeLa
D8		GSE83438 (Gokhale et al., 2016)	2,527			Huh7
D9		GSE78030 (Patil et al., 2016)	20,694			HEK293T
D10	YTHDF2	GSE49339 (Wang et al., 2014)	22,688	28,970	6,677	HeLa
D11		GSE83438 (Gokhale et al., 2016)	5,147			Huh7
D12		GSE78030 (Patil et al., 2016)	6,280			HEK293T
D13	YTHDF3	GSE86214 (Shi et al., 2017)	2,608	7,253	3,495	HeLa
D14		GSE83438 (Gokhale et al., 2016)	177			Huh7
D15		GSE78030 (Patil et al., 2016)	5,082			HEK293T
D16	EIF3A	GSE65004 (Lee et al., 2015)	45	756	470	HEK293T
D17		GSE73405 (Meyer et al., 2015)	731			

### Feature Encoding Scheme and Selection

We considered both the conventional sequence-derived features and the genome-derived features.

The sequence-derived features were summarized in the iLearn (Chen Z. et al., 2019; Chen et al., 2020) and BioSeq-Analysis (Liu, 2019; Liu et al., 2019), which can be divided into six different classes. Based on their classification, we chose one method from each class including nucleic acid composition (Lee et al., 2011), binary encoding method (Wu et al., 2015), position-specific tendencies of trinucleotide (He et al., 2018), electron-ion interaction pseudopotentials (He et al., 2019), Autocorrelation and pseudo k-tupler composition (Liu et al., 2015). Also, the chemical property combined with nucleic frequency, which is a popular encoding method in recent years (Bari et al., 2013; Chen et al., 2016a, b, 2017a; Li et al., 2018), was also used in performance testing for m^6^A reader target prediction.

The genomic features shown in previous projects (Chen K. et al., 2019; Song et al., 2019) are effective in RNA modification prediction. In order to improve the performance of the predictor, 58 mammalian genome features belonging to 9 classes were applied. All the features used were generated by the “GenomicFeatures R/Bioconducter” package using the transcript annotations hg19 TxDb package (Lawrence et al., 2013). The first class involves dummy variables indicating whether the adenosine site overlaps the topological region within the RNA transcript. The second class specifies the relative position of the adenosine site on the region, while the third class tells the length of the target mRNA transcript. Features belonging to the fourth class measure the nucleotide distances to the splicing junction and the nearest neighboring site. The fifth and sixth classes are based on clustering information of modification sites and scores related to conservation (Siepel et al., 2005; Gulko et al., 2015), respectively. The last three feature groups describe RNA secondary structures (Lorenz et al., 2011), genomic properties and attributes of the genes or transcripts, respectively. More details of the genomic features considered in our analysis were presented in Supplementary Table S1.

### Feature Selection Technique

With multiple features, the dimension of dataset increases, leading to overfitting, information redundancy or increased computational time. To solve this problem, feature selection is effective in optimizing relevant modeling variables and improving the accuracy of the constructed models. In this study, we performed feature selection using F-score technique (Lin et al., 2014; Dao et al., 2019). Technically, F-score is a wrapper-type feature selection algorithm, used to measure the degree of difference between two real-number data sets. For a given training sample *x*_*d*_, there are *n*^+^ positive samples and *n*^−^ negative samples. The *F*-score for the i-th feature can be calculated as:

$$F_{i}=\frac{{\left({\bar{x}}_{i}^{\left(+\right)}-{\bar{x}}_{i}\right)}^{2}+{\left({\bar{x}}_{i}^{\left(-\right)}-{\bar{x}}_{i}\right)}^{2}}{\frac{1}{n^{+}-1}\,\sum_{k=1}^{n^{+}}{\left({\bar{x}}_{d,i}^{\left(+\right)}-{\bar{x}}_{i}^{\left(+\right)}\right)}^{2}+\frac{1}{n^{-}-1}\,\sum_{d=1}^{n^{+}}{\left({\bar{x}}_{d,i}^{\left(-\right)}-{\bar{x}}_{i}^{\left(-\right)}\right)}^{2}}$$

where ${\bar{x}}_{i}^{\left(+\right)}$, ${\bar{x}}_{i}^{\left(-\right)}$ and ${\bar{x}}_{i}$ denote the average frequency of the i-th feature in the positive, negative and the whole samples, respectively; ${\bar{x}}_{d,i}^{\left(+\right)}$ and ${\bar{x}}_{d,i}^{\left(-\right)}$ represent the value of the i-th feature of the d-th sequence in the positive and negative samples, respectively. A larger F-score value means better predictive ability of a feature. To demonstrate this relative distinguishing ability of every genomic feature, the computed *F*-score values were rescaled between 0 and 1, and ranked in the descending order. Referring to this ranking, we used incremental feature selection (IFS) and SVM method to complete the selection process (Chen and Lin, 2006; Lin et al., 2014). Specifically, the feature subset begins with the feature with the highest *F*-score, and the next feature subset contains the last feature subset and one next feature. AUC values of 5-fold cross-validation were obtained for each feature subset.

### Machine Learning Approach and Performance Evaluation

To reduce the bias in the experiment, especially when selecting the polyA RNAs during library preparation, we built separate prediction models using full transcript data and mature mRNA data, respectively. In the mature mRNA predictor, only m^6^A sites located in exon regions are considered.

Since the positive-to-negative ratio of our datasets was highly unbalanced (1:10), we randomly split the negative data into ten parts and combined with the positive dataset with 1:1 positive-to-negative ratio to avoid the unfavorable choice of machine learning classifiers. Subsequently, 10 models were trained and the average outcome score was reported as the performance of the classifier. For each m^6^A reader, the target sites identified in different experiments were mixed, and then the predictor was trained with 80% of the total sites before being evaluated by the remaining 20% of sites for independent testing. Specifically, the mature mRNA datasets for YTHDF1-3, YTHDC1-2, EIF3a have 39577, 44025, 11065, 24312, 1245, and 1200 training data, and 9895, 11007, 2767, 6078, 311, and 300 testing data. The full transcript datasets for those m^6^A readers have 40955, 46352, 11605, 26662, 1970, and 1210 training data, and 10239, 11588, 2901, 6666, 492, and 302 testing data.

Machine learning algorithms have been widely applied in many fields of biological research such as predicting structural and functional properties of biological sequences. We applied Support Vector Machine (SVM) (Chang and Lin, 2011) to compare encoding schemes and approaches. To identify a better algorithm for model construction, we compared multiple machine learning algorithms including SVM, Logistic Regression (LR), Random Forest (RF), and XGBoost.

To validate the model performance, besides 5-fold cross-validation, we also applied the cross-sample test, in which the sites reported from one sample (or condition) were reserved for testing purpose and the sites reported in all other samples (or conditions) were used for training. This testing mode directly evaluates the capability of the prediction approach to detect reader-specific target sites under a single biological condition not profiled previously. Besides, four commonly used performance metrics are used for performance evaluation, including Area under the ROC Curve (AUC) (Bradley, 1997), Precision-Recall Curve (PR AUC) (Keilwagen et al., 2014), accuracy (Acc) (Jin and Ling, 2005) and Mathew’s correlation coefficient (MCC) (Powers, 2008). The formula of Acc and MCC are as follows:

$$\begin{array}{c} Acc=\frac{TP+TN}{TP+FN+TN+FP}\\ MCC=\frac{TP\times TN-FP\times FN}{\sqrt{\left(TP+FP)\times (TP+FN)\times (TN+FP)\times (TN+FN\right)}} \end{array}$$

where TP is the number of true positives, TN the number of true negatives, FP the number of false positives and FN the number of false negatives.

Model construction and performance evaluation were conducted in R (Version 3.6.3). Machine learning algorithms were supported by caret package (Kuhn, 2020).

## Results and Discussion

### Feature Selection

Due to the high reliability and effectiveness in reflecting intrinsic relation to the targets, sequence-derived features have been widely used and achieved high accuracy in extensive researches focusing on the m^6^A site prediction. However, genome-derived features have been discovering and currently showing a new perspective in feature extraction (Zhou et al., 2016; Chen et al., 2017a). Here, we extracted genome features from 41 bp sequence data. We employed WHISTLE approach to combine both sequence-derived features and genome-derived features to predict the target specificity of m^6^A readers. To increase robustness and reduce overfitting of the predicter, feature selection was performed, where those most relevant features to the targets were identified.

Initially, all the genomic features were normalized to ensure the equal contribution of each feature. Then the *F*-score method was applied to allow all features to be ranked accordingly. Combining IFS and SVM, AUC value of 5-fold cross-validation were obtained for each feature subset. By examining AUC scores, the best performance was achieved by the optimal feature subset. The detailed feature selection results were summarized in Supplementary Figures S1–S6 for YTHDF1-3, YTHDC1-2 and EIF3A under both the full transcript and mature mRNA transcript, respectively. For example, it can be observed in Supplementary Figure S6A that, the best performance of EIF3A target prediction was achieved with the top 44 features for the mature mRNA model. Therefore, only the top 44 features were used ultimately to build the mature mRNA prediction models for EIF3A target prediction. Likewise, feature selection in target prediction was conducted for every other reader, and the predictors were constructed in the same way.

### Performance Based on Different Features

With the nucleotide encoding methods based on chemical properties, extensive studies have achieved high accuracy in the m^6^A site prediction. However, for the first time, we explored and compared different sequence encoding schemes for predicting the target specificity of m^6^A-binding proteins.

For each m^6^A reader, the target sites identified in different experiments were mixed, and then the predictor was trained with 80% of the total sites before being evaluated by the remaining 20% of sites for independent testing. As a comparison, the performance of 5-fold cross-validation on the training data was also reported. As shown in Supplementary Table S7, m^6^A reader achieved AUC scores of 0.981 and 0.893 in independent testing under the full transcript and mature mRNA models, respectively. This performance is substantially better than other approaches that did not take advantage of genome-derived features.

Subsequently, we evaluated the capability of the proposed method in identifying the reader-specific target m^6^A sites under different biological contexts. In this test, the sites generated from each sample were used for independent testing, while all other samples were used for training, so the training sites and the test sites were not reported from the same condition. This is often the real scenario of interest where models are constructed to predict target sites in a new biological context. Besides this cross-condition test, the results of 5-fold cross-validation on the training data were also presented. The detailed evaluation results on every individual sample for every reader are shown in Supplementary Tables S2–S6, with a summary of the cross-condition tests presented in Table 3. It can be seen that our approach achieved a high accuracy with AUC scores of 0.975 and 0.873 under full transcript and mature mRNA models in the cross-condition test. The performance is again substantially better than the competing methods.

**TABLE 3:** Target prediction performance under cross-condition test.

Mode	Method	YTHDC1	YTHDC2	YTHDF1	YTHDF2	YTHDF3	EIF3A	Average
Full transcript model	m^6^A reader	0.974	0.920	0.983	0.983	0.992	1.000	0.975
	Composition	0.769	0.713	0.773	0.778	0.782	0.893	0.785
	MethyRNA	0.763	0.611	0.795	0.794	0.787	0.849	0.767
	EIIP	0.770	0.713	0.768	0.778	0.782	0.894	0.784
	PseKNC	0.733	0.643	0.743	0.755	0.753	0.852	0.747
	AutoCo	0.651	0.586	0.673	0.684	0.737	0.835	0.694
	PSNP	0.777	0.654	0.816	0.816	0.894	0.869	0.804
	onehot	0.750	0.603	0.796	0.795	0.791	0.858	0.766
Mature mRNA model	m^6^A reader	0.815	0.730	0.983	0.839	0.883	0.987	0.873
	Composition	0.660	0.503	0.773	0.667	0.707	0.872	0.697
	MethyRNA	0.659	0.631	0.795	0.695	0.733	0.833	0.724
	EIIP	0.670	0.504	0.768	0.667	0.727	0.871	0.701
	PseKNC	0.635	0.593	0.743	0.630	0.706	0.837	0.691
	AutoCo	0.527	0.556	0.673	0.559	0.688	0.820	0.637
	PSNP	0.703	0.675	0.816	0.754	0.858	0.870	0.779
	onehot	0.662	0.622	0.796	0.696	0.757	0.836	0.728

*In this test, the sites generated from each sample were used for independent testing, while all other samples were used for training, so the training sites and the test sites were not reported from the same condition. This is often the real scenario of interest where models are constructed to predict target sites under a new biological context. See Supplementary Tables S2–S6 for more detailed results.*

### Detect Potential Substrate of m^6^A Readers

**FIGURE 1:**
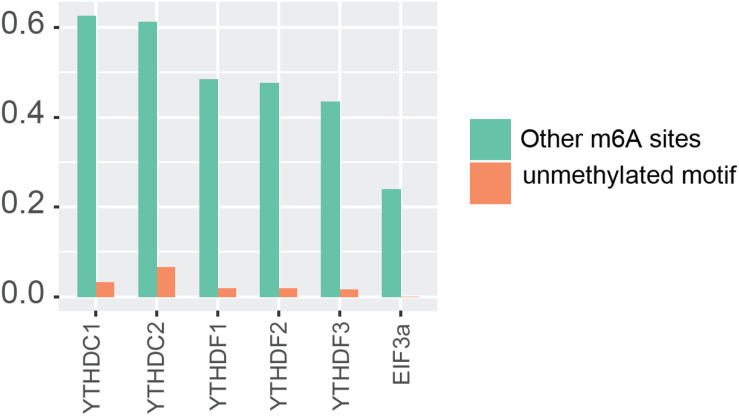
Potential substrate of m^6^A readers.

**FIGURE 2:**
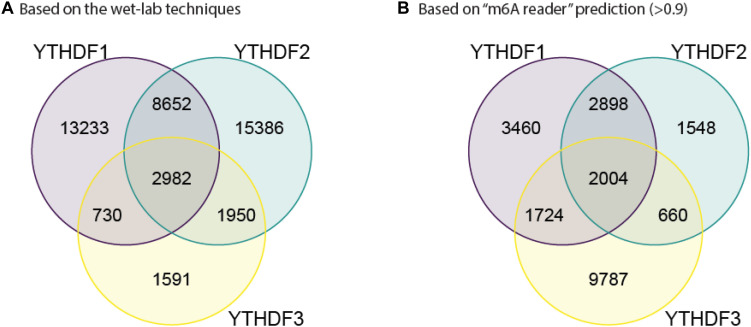
Substrate overlap between YTHDF family.

In order to further confirm the reliability and efficiency of our predictors, we used our predictors to detect m^6^A reader binding sites on the unidentified regions. As expected, all m^6^A readers bind to more than 20% m^6^A sites, while they bind to less than 10% unmethylated motifs as shown in Figure 1. The binding preference is significant and reasonable, which demonstrated the high discrimination ability of our predictors. Moreover, we compared the previous binding sites of YTHDF family (Figure 2A) and the prediction result of them on unidentified regions (Figure 2B). The wet-lab and prediction result shows that readers in YTHDF family have both common and distinct binding sites, suggesting that the binding sites of YTHDF proteins are not exactly identical. This is not consistent with the conclusion in the previous study that YTHDF proteins bind to identical sites on all m^6^A mRNAs (Zaccara and Jaffrey, 2020). Our result suggests that YTHDF family proteins have similar functions of mediating degradation of m^6^A mRNAs, and they also have different functions in mRNA regulation simultaneously. This result is consistent with our GO enrichment analysis, and also partially supports that m^6^A readers’ effect on downstream processes are much more heterogeneous and context-dependent across transcripts (Zhang et al., 2020). The predicted probabilities for the targeting of each m^6^A reader are provided on the download page of the website^1^.

### Model Comparison

To discover a better machine learning algorithm for our proposed models, we compared the performance of SVM, LR, RF, and XGBoost on mature mRNA and full transcript data for the prediction of target specificity of six m^6^A readers. In general, the performances of different machine learning algorithms are all very high (>0.8 for mature mRNA models and >0.9 for full transcript models) and have little difference among them as shown in Supplementary Table S8. Therefore, we decided to use SVM classifier for the predictors.

### Characterizing the Target Specificity of m^6^A Readers

Our result suggests that the substrates of m^6^A readers can be classified, reflecting the distinct biological characteristics of each m^6^A reader. We thus explored the distribution, conservation, and functional relevance of the substrates of each m^6^A reader.

**FIGURE 3:**
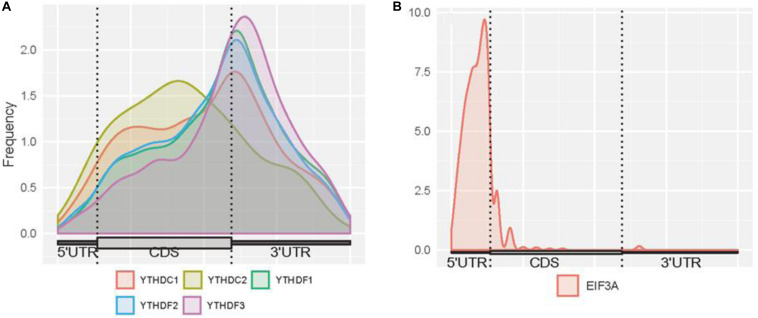
Distribution of m^6^A readers binding sites on mRNAs. **(A)** Distribution of the binding sites of YTHDC1, YTHDC2, YTHDF1, YTHDF2, and YTHDF3 on mRNAs. **(B)** Distribution of the binding sites of EIF3A on mRNAs. The figures were plotted using the Guitar R/Bioconductor package (Cui et al., 2016).

Here, we firstly examined the distribution of binding sites for each reader (Figure 3). High enrichment of YTHDC1 is observed around stop codons and CDSs. However, it can be noticed that the binding abundance of YTHDC1 is relatively lower than members of YTHDF family in stop codons, while it is highly enriched in CDSs. This is consistent with the fact that YTHDC1 is not only targeting to m^6^A sites at its C terminus but also directly interacting with pre-mRNA splicing factor SRSF3 or SRSF10, which prefers to reside on the upper stream of m^6^A sites (Roundtree et al., 2017). The spatial association among those proteins implicates the process of recruiting pre-mRNA splicing factors and inducing mRNA splicing outcomes. Surprisingly, YTHDC2 targets are more enriched in CDSs near stop codons than in 3′ UTR, suggesting that YTHDC2 is distinct from other m^6^A readers. As YTHDC2 is reported to be the largest protein (∼160 kDa) among all YTH family members and with numerous RNA binding domains (e.g., helicase domain and two Ankyrin repeats, Hsu et al., 2017) apart from YTH domain, besides its acknowledged functions of accelerating translation and degradation of mRNAs as an m^6^A reader, it is possible that there are potential underlying functions independent from m^6^A-binding remained to be discovered. For instance, the recent study indicated that YTHDC2 as an RNA induced ATPase moves along the RNA from 3′ to 5′ with helicase activity, and interacts with 5′ to 3′ exoribonuclease XRN1 mediated by two Ankyrin repeats (ANK) on YTHDC2 (Wojtas et al., 2017). Remarkably, YTHDF family shows a similar binding distribution in CDSs and 3′ UTRs with peaks at around stop codons of mRNAs. A similar pattern of results was obtained in previous studies suggesting that YTHDFs directly interplay among one another to collaboratively regulate translation and decay of targeted mRNAs in the cytoplasm (Shi et al., 2017). The binding sites of EIF3A are uniquely enriched at 5′UTRs. This is directly in line with previous findings that the HLH motif of EIF3A interacts predominantly with the m^6^A residues on the 5′UTR, and EIF3A specifically functions to promote cap-independent translation under diverse cellular stresses.

**FIGURE 4:**
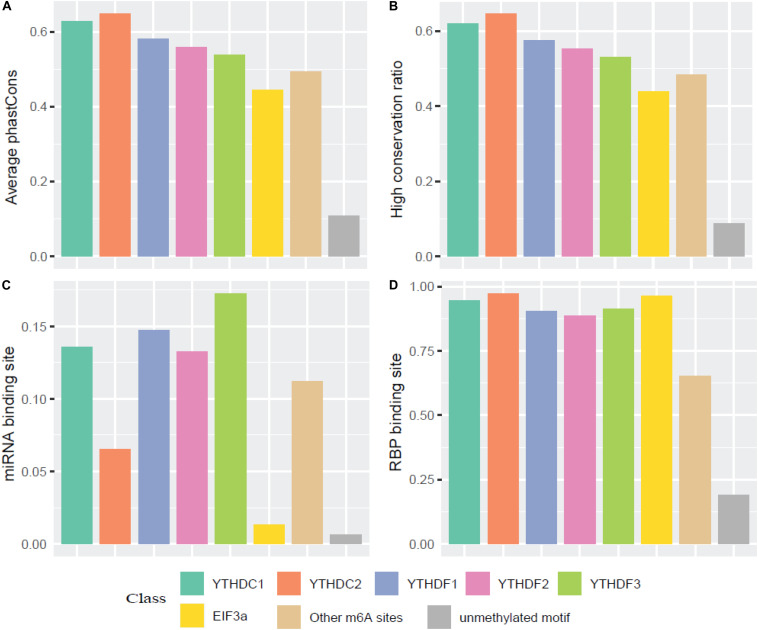
Conservation analysis of the m^6^A sites targeted by different readers. **(A)** Average phastCons; **(B)** High conservation ratio; **(C)** Frequency of miRNA binding site among the targets of six m^6^A readers; **(D)** Frequency of RBP binding site among the targets of six m^6^A readers.

We then compared the conservation of all m^6^A readers by phastCons score and high conservation ratio (>0.5). As seen in Figures 4A,B, the m^6^A sites (targeted or not targeted by the studied six readers) are more conservative than unmethylated m^6^A motifs (DRACH). This suggests that m^6^A sites and the m^6^A reader binding sites are more evolutionarily conserved at the gene level, and the occurrence of m^6^A should be considered of functional importance and maintained under selection pressure. Moreover, the YTH family is more conserved compared with other regulation components, which is similar to the finding that YT521-B homology (YTH) RNA-binding domain in eukaryotes is known to be highly conserved with essential Lys-364, Trp-380, and Arg-478 (Zhang et al., 2010). Additionally, as shown in Figure 4C, compared with EIF3a binding sites and unmethylated sites which are mostly not in 3′ UTR, targets of other m^6^A readers and other untargeted m^6^A sites are more correlated with the miRNA binding sites. This result agrees well with existing studies investigated that miRNA targets are more enriched in 3′ UTR and m^6^A peaks prior to the present of miRNA binding for a majority of the time, suggesting that m^6^A modification functions to enhance initiation of miRNA biogenesis (Meyer et al., 2012; Alarcón et al., 2015). And the relative low overlapping rate between YTHDC2 binding sites and miRNA binding sites could be explained by multiple RNA-binding domains of YTHDC2. Furthermore, the proportions of overlapping of RNA-binding proteins (RBPs) and each m^6^A reader’s binding site are calculated. Figure 4D shows that RBPs binding regions overlap with m^6^A reader binding sites in mRNA more than the other m^6^A sites, while there are even fewer overlapping regions with unmethylated sites. This is consistent with our knowledge that some RBPs are essential in post-transcriptional control of RNAs including splicing, stabilization, localization and translation of mRNA. In the process of regulating transcription and translation, m^6^A readers may recruit large numbers of regulators or factors to their targeted RNAs so as to functionally regulate biological processes (Shi et al., 2017).

**FIGURE 5:**
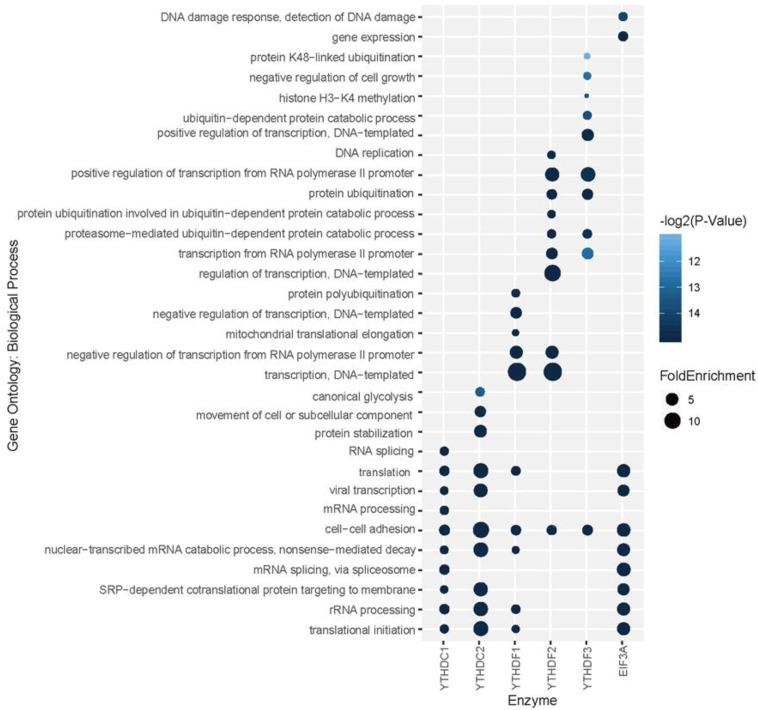
Gene ontology (GO) enrichment analysis for each reader’s substrates. The top 10 GO functions related to each m^6^A readers are presented.

To explore the association among m^6^A modification, readers and biological functions, the gene ontology (GO) enrichment analysis was conducted to measure the biological functions of substrates of each reader using DAVID websites (Huang da et al., 2009). The resulting top 10 GO functions related to each m^6^A readers were illustrated in Figure 5. Interestingly, YTHDC1 is involved in mRNA splicing, mRNA processing and nuclear-transcribed mRNA catabolic process, which is consistent with our understanding of its role of mediating nuclear to cytoplasmic export of nascent m^6^A-containing mRNAs (Roundtree et al., 2017). The targeting of YTHDC2, shown to accelerate the degradation of mRNA and enhance translation efficiency (Hsu et al., 2017), are more related to nonsense-mediated decay, protein stabilization and translational initiation. YTHDF1 targets are enriched under the GO terms of nuclear-transcribed mRNA catabolic process and translation initiation (Wang et al., 2015), suggesting its function in selectively recruiting of ribosomes and facilitating translation. YTHDF2 and YTHDF3 targets are both associated with proteasome-mediated ubiquitin-dependent protein catabolic process, which corresponds to our knowledge of their regulation in the metabolism of cytosolic m^6^A-modified mRNAs (Wang et al., 2014; Shi et al., 2017). EIF3A, reported to serve as a driver of specialized translation (Lee et al., 2015), is enriched with gene expression, translation and SRP-dependent co-translational protein targeting to the membrane. Moreover, as summarized in Supplementary Figure S7, six m^6^A readers show high enrichment in cytosol, cytoplasm, and membrane. Five of them (YTHDC1, YTHDF1-3, and EIF3a) are enriched in nucleus and nucleoplasm. While YTHDC2 is more enriched in extracellular exosome, extracellular matrix and myelin sheath instead of nucleus or nucleoplasm. All six proteins are enriched in the function of protein binding and poly(A) RNA binding, while they each have other specialized functions. This is consistent with analysis above on the enrichment of biological process and previous relevant literature. All gene ontology enrichment results were shown in Supplementary Table S9.

**FIGURE 6:**
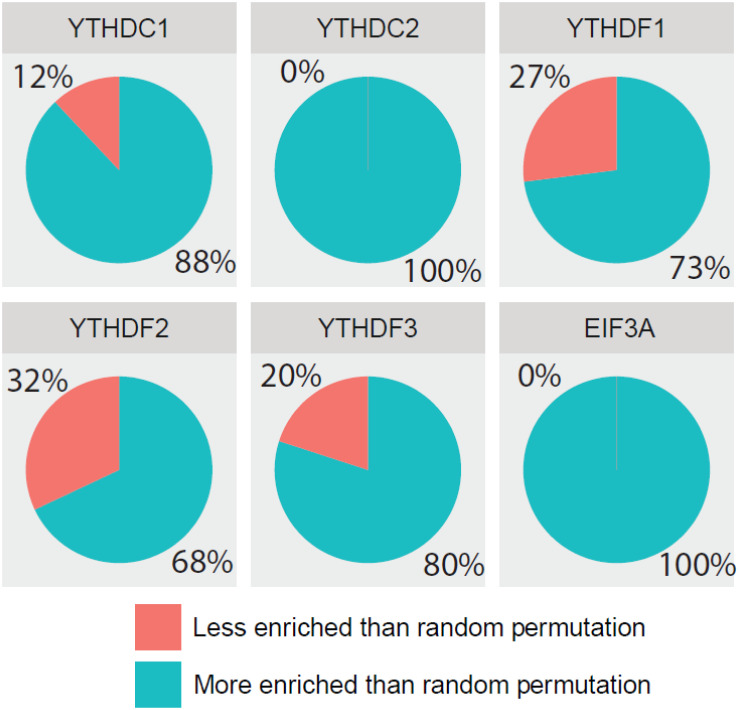
Comparing detection of m^6^A readers’ targets based on biological significance. The most significant *p*-values of top 10 GO terms treated with negative logarithm were added up, and those results of identified targets were compared with those of randomly selected substrates. With the bootstrap sampling approach, substrates were randomly selected and analyzed for 100 times before the results were summarized as proportions and displayed in pie charts.

Additionally, we further confirmed the biological meanings of the substrates of all m^6^A readers. Based on the results of previous GO enrichment analysis (Chen K. et al., 2018), the most significant *p*-values of top 10 terms treated with the negative logarithm were firstly added up, and then those computed results of identified targets were compared with those of randomly selected substrates. With the bootstrap sampling approach, substrates were randomly selected and analyzed for 100 times before the results were summarized as proportions and displayed in pie charts. Conceivably, if our results achieved on real data are more biologically meaningful than random permutation, it is possible that our analysis reliably unveiled the true biological functions. Specifically, there are 88, 100, 73, 68, 80, and 100% chances for each reader to be more enriched in biological functions than random permutation as illustrated in Figure 6, suggesting high possibility that our functional prediction for each individual reader is statistically meaningful.

### Web Sever for m^6^A Reader

**FIGURE 7:**
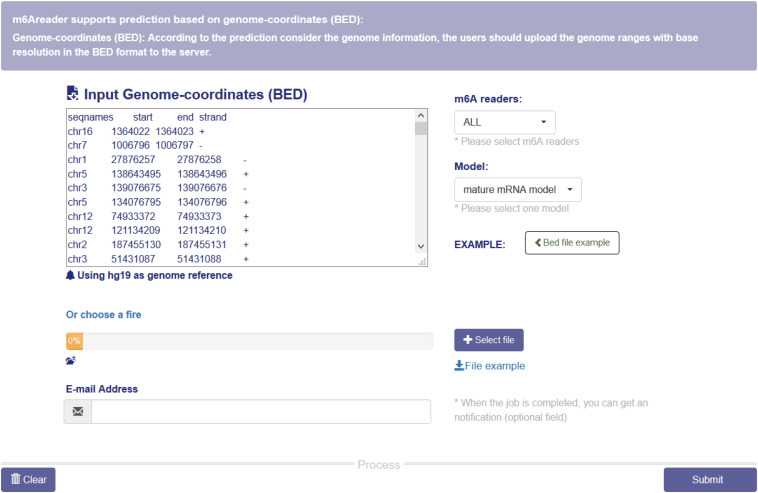
m^6^A reader web server. The web server takes genome ranges in BED format as the input, and supports prediction for the target sites of six m^6^A readers (YTHDC1, YTHDC2, YTHDF1, YTHDF2 and YTHDF3 and EIF3A). All the materials used in the project, including the training data and codes, are also available on the website.

A web server with a friendly graphical user interface (Figure 7) was constructed to properly share the predictive models we constructed for predicting target specificity of the m^6^A readers. Users may upload the genome ranges in BED format to the website, and a notification email will be sent to the given email address once the job is finished.

## Conclusion

With the great breakthroughs made in RNA modification-mediated regulation of gene expression, studies of emerging transcriptome modifications have driven rapid development of the high-throughput sequencing technologies. With the aid of the invention of m^6^A-seq (Dominissini et al., 2012) and MeRIP-seq (Meyer et al., 2012), transcriptome-wide profiling of m^6^A is now possible. Based on comprehensive high-throughput sequencing data, MeT-DB (Liu H. et al., 2018) and RMBase (Xuan et al., 2018) were established, providing the site information of RNA modifications. Subsequently, single-based technologies such as m^6^A-CLIP (Ke et al., 2015) and miCLIP (Linder et al., 2015) were also developed to precisely identify the positions of m^6^A. Complementary to experimental methods, well-established computational models facilitate the analysis of sequencing data and address the challenges presented in the bioinformatics community by predicting potential RNA methylation sites. The exomePeak R/Bioconductor package (Meng et al., 2013, 2014), MACS algorithm (Zhang et al., 2008) and DRME software (Liu et al., 2016) were introduced to analyze epitranscriptome profiling data, which improved our understanding of RNA methylation. Sequence-based site prediction models such as iRNA(m^6^A)-PseDNC (Chen W. et al., 2018) and iRNAMethyl (Chen et al., 2015b) applied statistical methods, whereas m^6^Apred (Chen et al., 2015c), RAM-ESVM (Chen et al., 2017b), and RNAMethPre (Xiang et al., 2016) integrated machine learning approaches, predicting m^6^A sites in different species’ transcriptome. Furthermore, potential RNA methylation-disease associations have been revealed by m^6^Avar (Zheng et al., 2018) and m^6^ASNP (Jiang et al., 2018). With a similar purpose, heterogeneous networks have been used in DRUM (Tang et al., 2019), FunDMDeep-m^6^A (Zhang et al., 2019b) and Deepm^6^A (Zhang et al., 2019a), showing a new perspective in studying disease-associated RNA methylation.

In this study, we constructed SVM-based models for the prediction of target specificity of m^6^A readers (YTHDC1, YTHDC2, YTHDF1, YTHDF2, YTHDF3, and EIF3A). The proposed models rely on 58 genomic features integrated with the sequence features related to chemical properties. After feature selection using the *F*-score method, those models achieved high prediction performance in 5-fold cross-validation and independent testing. Additionally, we compared the performance of different sequence encoding schemes on each reader’s substrate prediction. As existing m^6^A base-resolution data suffer from the bias of polyA selection, mature mRNA model was also considered besides the full transcript model. Moreover, we compared different machine learning algorithms and showed that four algorithms all demonstrate high performance with little difference in the prediction of target specificity of m^6^A readers. We eventually decided to use SVM classifier for our predictors.

It is also worth mentioning that our comprehensive analysis of m^6^A readers revealed potential regulatory patterns and biological relationships. We showed that m^6^A reader binding sites on mRNAs were concentrated in CDSs and 3′ UTR near stop codons, which is in line with m^6^A localization. Although distribution analysis of m^6^A readers has been conducted in previous studies and suggested similar binding patterns (Xu et al., 2014; Wang et al., 2015; Hsu et al., 2017), the results we presented were substantially enhanced with the incorporation of multiple datasets. Our result shed lights on the post-transcriptional and translational functions of m^6^A readers on m^6^A-containing mRNAs with more reliable evidence. Moreover, computed phastCons score and conservation ratio revealed a high conservation of the target sites of m^6^A readers, suggesting that they are possibly playing necessary or essential roles in regulating m^6^A-containing mRNAs. This is remarkable since we focused on the conservation of binding sites of m^6^A readers on mRNAs, rather than the conservation of m^6^A motifs itself as widely studied currently (Meyer et al., 2012), thus the biologically meaningful relationship between m^6^A readers and m^6^A modifications was confirmed. Besides, different from enrichment analysis alone in previous studies (Hsu et al., 2017), we not only unveiled functional relevance through the enrichment of the targets of m^6^A readers in biological process, cellular components and molecular functions by GO analysis, but also confirmed that reader-regulated sites are more likely to be biologically significant than randomly selected sites. The combination of statistical analysis and GO analysis ensures the robust detection and critical evaluation of the biological functions with a higher degree of confidence. Furthermore, our GO enrichment analysis result is also consistent with the wet-lab experiment and our prediction on unidentified regions that YTHDF proteins have both similar functions and different functions in the m^6^A mRNA regulation. This supports the conclusion made in previous study that m^6^A readers’ effect on downstream processes are much more heterogeneous and context-dependent across transcripts (Zhang et al., 2020).

However, this study has a number of limitations that could be improved in the future. Firstly, it has been argued that 4SU PAR-CLIP suffers from U-bias in contrast with UV-254 crosslinking or 6SG crosslinking (Ascano et al., 2012), thus other CLIP techniques are recommended to ensure crosslinking efficiency. Secondly, although data from different experiments were combined to build the predictors and 5-fold cross-validation was used to balance the bias-variance tradeoff, data of YTHDC2 and EIF3A substrates are still limited, which may make overfitting of the models possible. Thus, the analysis and prediction will benefit from other data from wet experiments in the future. Thirdly, as genome-derived features improved the performance of predictors dramatically, this suggests that genomic features carry important characteristics of biological data. Considering only 58 of them were involved in the feature selection procedure, it is worth exploring more genomic features so as to allow more effective features to be selected and reduce the bias as much as possible. In the future, it is expected to see the expanded studies of the enzyme target specificity and functional associations of other RNA modifications, such as m^1^A and Pseudouridine, on other types of RNAs, such as lncRNA and snRNAs, and in other species, such as mouse and yeast. Additional studies are clearly needed to investigate RNA-sequence-dependent m^6^A readers other than YTH domain-containing proteins such as FMR1 (Edupuganti et al., 2017). And it could be quite interesting to explore disease-associated RNA modification based on cellular binding patterns of regulatory proteins on modified RNAs.

## Data Availability Statement

The datasets presented in this study can be found in online repositories. The names of the repository/repositories and accession number(s) can be found in the article/ Supplementary Material.

## Author Contributions

KC conceived the idea, initialized the project, collected and processed the training and benchmark datasets. ZW generated the genomic features. DZ, YW, YZ, and HX built machine learning models. YT and BS designed and built the web server. DZ, YW, and YZ drafted the manuscript. All authors read, critically revised, and approved the final manuscript.

## Conflict of Interest

The authors declare that the research was conducted in the absence of any commercial or financial relationships that could be construed as a potential conflict of interest.

## Footnotes

1 http://m6Areader.rnamd.com
