# Supplementary material for: m6A Reader: Epitranscriptome Target Prediction and Functional Characterization of N6-Methyladenosine (m6A) Readers
Source: Front Cell Dev Biol. 2020 Aug 11;8:741. doi: 10.3389/fcell.2020.00741 (PMC7431669; doi:10.3389/fcell.2020.00741)
Supplement: Supplementary file 2 [file Table_2.DOCX]

**Table S1 Genomic features used in the analysis**

| ID | Name | Description | Note |
| --- | --- | --- | --- |
| 1 | UTR5 | 5' UTR | Dummy variables indicating whether the site is overlapped to the topological region on the major RNA transcript. |
| 2 | UTR3 | 3' UTR |  |
| 3 | cds | CDS |  |
| 4 | Stop_codons | stop codons flanked by 100bp |  |
| 5 | Start_codons | start codons flanked by 100bp |  |
| 6 | TSS | downstream 100bp of TSS |  |
| 7 | TSS_A | downstream 100bp of TSS on A |  |
| 8 | exon_stop | exons containing stop codons |  |
| 9 | alternative_exon | alternative exons |  |
| 10 | constitutive_exon | constitutive exons |  |
| 11 | internal_exon | Internal exons |  |
| 12 | long_exon | long exons (exon length >= 400bp) |  |
| 13 | last_exon | last exons |  |
| 14 | last_exon_400bp | 5’ 400bp of the last exons |  |
| 15 | last_exon_sc400 | 5’ 400bp of the last exons containing stop codons |  |
| 16 | intron | introns |  |
| 17 | pos_UTR5 | relative position on 5'UTR | Relative position on the region |
| 18 | pos_UTR3 | relative position on 3'UTR |  |
| 19 | pos_cds | relative position on CDS |  |
| 20 | pos_exons | relative position on exon |  |
| 21 | dist_sj_5_p2000 | distance to the upstream (5' end) splicing junction | The region length in bp. |
| 22 | dist_sj_3_p2000 | distance to the downstream (3' end) splicing junction |  |
| 23 | length_UTR3 | 5'UTR length |  |
| 24 | length_UTR5 | 3'UTR length |  |
| 25 | length_cds | CDS length |  |
| 26 | length_gene_ex | mature transcript length |  |
| 27 | length_gene_full | full transcript length |  |
| 28 | length_tx_exon | transcript exonic length |  |
| 29 | length_tx_full | transcript exonic length |  |
| 30 | clust_DRACH_f1000 | number of DRACH motif neighboors within 1000bp flanking regions | Clustering information |
| 31 | clust_DRACH_f100 | number of DRACH motif neighboors within 100bp flanking regions |  |
| 32 | dist_DRACH_p2000 | distance to the nearest DRACH motif (peaked at 2000bp) |  |
| 33 | dist_DRACH_p200 | distance to the nearest DRACH motif (peaked at 200bp) |  |
| 34 | PC_1bp | phastCons scores of the nucleotide | Scores related to evolutionary conservation |
| 35 | PC_101bp | average phastCons scores within the flanking 50bp region |  |
| 36 | FC_1bp | fitCons scores of the nucleotide |  |
| 37 | FC_101bp | average fitCons scores within the flanking 50bp region |  |
| 38 | struct_hybridize | Predicted RNA hybridized region | RNA secondary structure |
| 39 | struct_loop | Predicted RNA loop region |  |
| 40 | HNRNPC_eCLIP | eCLIP data of HNRNPC RNA binding sites | RNA binding protein annotation from MeTDB database |
| 41 | miR_targeted_genes | miRNA targeted genes |  |
| 42 | TargetScan | miRNA targeted sites verified by experiment[8] |  |
| 43 | Verified_miRtargets | Predicted miRNA targeted sites by TargetScan |  |
| 44 | METTL3_TREW | METTL3 binding region |  |
| 45 | METTL14_TREW | METTL14 binding region |  |
| 46 | WTAP_TREW | WTAP binding region |  |
| 47 | METTL16_CLIP | METTL16 binding region |  |
| 48 | ALKBH5_PARCLIP | ALKBH5 binding region |  |
| 49 | FTO_CLIP | FTO binding region |  |
| 50 | FTO_eCLIP | FTO binding region (eCLIP) |  |
| 51 | sncRNA | sncRNA | Gene characteristic |
| 52 | lncRNA | lncRNA |  |
| 53 | isoform_num | isoform number |  |
| 54 | exon_num | exon number |  |
| 55 | HK_genes | housekeeping genes |  |
| 56 | GC_cont_genes | gene level GC content z score |  |
| 57 | GC_cont_101bp | 101bp GC content z score |  |
| 58 | GC_cont_101bp_abs | 101bp GC content z score |  |

**Table S2 Performance of YTHDC1 target prediction using cross-condition test**

|  | Encoding Method | Full transcript model | | | | | Mature mRNA model | | | | |
| --- | --- | --- | --- | --- | --- | --- | --- | --- | --- | --- | --- |
|  |  | D1 | D2 | D3 | D4 | Average | D1 | D2 | D3 | D4 | Average |
| Cross  Validation | m6Areader | 0.975 | 0.974 | 0.976 | 0.969 | 0.974 | 0.810 | 0.805 | 0.813 | 0.771 | 0.801 |
|  | CONPOSITION | 0.757 | 0.746 | 0.754 | 0.831 | 0.769 | 0.587 | 0.623 | 0.560 | 0.679 | 0.608 |
|  | MethyRNA | 0.751 | 0.738 | 0.750 | 0.788 | 0.755 | 0.579 | 0.617 | 0.611 | 0.729 | 0.626 |
|  | EIIP | 0.757 | 0.747 | 0.753 | 0.831 | 0.769 | 0.587 | 0.622 | 0.560 | 0.703 | 0.611 |
|  | PseKNC | 0.714 | 0.703 | 0.710 | 0.773 | 0.723 | 0.601 | 0.578 | 0.598 | 0.721 | 0.620 |
|  | AutoCo | 0.572 | 0.600 | 0.590 | 0.640 | 0.599 | 0.527 | 0.526 | 0.525 | 0.545 | 0.530 |
|  | PSNP | 0.746 | 0.738 | 0.748 | 0.827 | 0.762 | 0.681 | 0.675 | 0.683 | 0.793 | 0.704 |
|  | onehot | 0.754 | 0.742 | 0.753 | 0.804 | 0.761 | 0.625 | 0.590 | 0.646 | 0.735 | 0.646 |
| Cross-condition Test | m6Areader | 0.981 | 0.977 | 0.966 | 0.971 | 0.974 | 0.854 | 0.839 | 0.784 | 0.757 | 0.815 |
|  | CONPOSITION | 0.817 | 0.804 | 0.771 | 0.696 | 0.769 | 0.608 | 0.742 | 0.727 | 0.575 | 0.660 |
|  | MethyRNA | 0.818 | 0.806 | 0.737 | 0.699 | 0.763 | 0.729 | 0.702 | 0.636 | 0.591 | 0.659 |
|  | EIIP | 0.817 | 0.804 | 0.771 | 0.696 | 0.770 | 0.683 | 0.743 | 0.699 | 0.575 | 0.670 |
|  | PseKNC | 0.778 | 0.767 | 0.737 | 0.668 | 0.733 | 0.692 | 0.691 | 0.637 | 0.555 | 0.635 |
|  | AutoCo | 0.699 | 0.650 | 0.652 | 0.610 | 0.651 | 0.553 | 0.526 | 0.517 | 0.513 | 0.527 |
|  | PSNP | 0.761 | 0.796 | 0.810 | 0.738 | 0.777 | 0.594 | 0.712 | 0.780 | 0.688 | 0.703 |
|  | onehot | 0.740 | 0.810 | 0.739 | 0.701 | 0.750 | 0.728 | 0.706 | 0.636 | 0.593 | 0.662 |

**Table S3 Performance of YTHDC2 and EIF3A target prediction using cross-condition test**

|  | Encoding Method | Full transcript model | | Mature mRNA model | |
| --- | --- | --- | --- | --- | --- |
|  |  | EIF3A | YTHDC2 | EIF3A | YTHDC2 |
| Cross  Validation | m6Areader | 0.995 | 0.946 | 0.959 | 0.983 |
|  | CONPOSITION | 0.857 | 0.857 | 0.779 | 0.732 |
|  | MethyRNA | 0.803 | 0.809 | 0.729 | 0.651 |
|  | EIIP | 0.858 | 0.859 | 0.779 | 0.733 |
|  | PseKNC | 0.853 | 0.808 | 0.776 | 0.642 |
|  | AutoCo | 0.838 | 0.696 | 0.758 | 0.522 |
|  | PSNP | 0.912 | 0.859 | 0.936 | 0.880 |
|  | onehot | 0.804 | 0.807 | 0.732 | 0.631 |
| Cross-condition Test | m6Areader | \ | \ | \ | \ |
|  | CONPOSITION | \ | \ | \ | \ |
|  | MethyRNA | \ | \ | \ | \ |
|  | EIIP | \ | \ | \ | \ |
|  | PseKNC | \ | \ | \ | \ |
|  | AutoCo | \ | \ | \ | \ |
|  | PSNP | \ | \ | \ | \ |
|  | onehot | \ | \ | \ | \ |

Note: According to the limitation of number of YTHDC2 and EIF3A target sites, testing on independent sample could not be performed for these two readers.

**Table S4 Performance of YTHDF1 target prediction using cross-condition test**

|  | Encoding Method | Full transcript model | | | | Mature mRNA model | | | |
| --- | --- | --- | --- | --- | --- | --- | --- | --- | --- |
|  |  | D7 | D8 | D9 | Average | D7 | D8 | D9 | Average |
| Cross  Validation | m6Areader | 0.981 | 0.985 | 0.985 | 0.983 | 0.844 | 0.861 | 0.863 | 0.856 |
|  | CONPOSITION | 0.827 | 0.776 | 0.770 | 0.791 | 0.765 | 0.661 | 0.575 | 0.667 |
|  | MethyRNA | 0.804 | 0.799 | 0.795 | 0.800 | 0.720 | 0.707 | 0.709 | 0.712 |
|  | EIIP | 0.826 | 0.749 | 0.770 | 0.770 | 0.760 | 0.658 | 0.650 | 0.690 |
|  | PseKNC | 0.753 | 0.735 | 0.728 | 0.739 | 0.697 | 0.616 | 0.610 | 0.641 |
|  | AutoCo | 0.632 | 0.676 | 0.669 | 0.659 | 0.536 | 0.553 | 0.554 | 0.548 |
|  | PSNP | 0.833 | 0.786 | 0.785 | 0.801 | 0.783 | 0.715 | 0.716 | 0.738 |
|  | onehot | 0.803 | 0.799 | 0.796 | 0.800 | 0.715 | 0.709 | 0.709 | 0.711 |
| Cross-condition Test | m6Areader | 0.983 | 0.983 | 0.983 | 0.983 | 0.836 | 0.865 | 0.849 | 0.850 |
|  | CONPOSITION | 0.731 | 0.773 | 0.814 | 0.773 | 0.615 | 0.722 | 0.715 | 0.684 |
|  | MethyRNA | 0.767 | 0.804 | 0.814 | 0.795 | 0.670 | 0.731 | 0.709 | 0.703 |
|  | EIIP | 0.731 | 0.771 | 0.802 | 0.768 | 0.615 | 0.719 | 0.716 | 0.683 |
|  | PseKNC | 0.712 | 0.747 | 0.769 | 0.743 | 0.591 | 0.667 | 0.667 | 0.642 |
|  | AutoCo | 0.653 | 0.675 | 0.692 | 0.673 | 0.540 | 0.560 | 0.567 | 0.556 |
|  | PSNP | 0.786 | 0.836 | 0.826 | 0.816 | 0.702 | 0.794 | 0.756 | 0.751 |
|  | onehot | 0.768 | 0.804 | 0.815 | 0.796 | 0.670 | 0.670 | 0.709 | 0.703 |

**Table S5 Performance of YTHDF2 target prediction using cross-condition test**

|  | Encoding Method | Full transcript model | | | | Mature mRNA model | | | |
| --- | --- | --- | --- | --- | --- | --- | --- | --- | --- |
|  |  | D10 | D11 | D12 | Average | D10 | D11 | D12 | Average |
| Cross  Validation | m6Areader | 0.981 | 0.982 | 0.983 | 0.982 | 0.847 | 0.850 | 0.850 | 0.849 |
|  | CONPOSITION | 0.763 | 0.831 | 0.809 | 0.801 | 0.650 | 0.751 | 0.717 | 0.706 |
|  | MethyRNA | 0.795 | 0.824 | 0.812 | 0.810 | 0.725 | 0.735 | 0.718 | 0.726 |
|  | EIIP | 0.763 | 0.830 | 0.810 | 0.801 | 0.655 | 0.751 | 0.751 | 0.707 |
|  | PseKNC | 0.710 | 0.790 | 0.790 | 0.711 | 0.608 | 0.706 | 0.673 | 0.662 |
|  | AutoCo | 0.635 | 0.695 | 0.686 | 0.672 | 0.534 | 0.581 | 0.569 | 0.561 |
|  | PSNP | 0.792 | 0.813 | 0.590 | 0.732 | 0.756 | 0.741 | 0.741 | 0.740 |
|  | onehot | 0.778 | 0.825 | 0.813 | 0.805 | 0.723 | 0.734 | 0.717 | 0.725 |
| Cross-condition Test | m6Areader | 0.980 | 0.984 | 0.984 | 0.983 | 0.814 | 0.848 | 0.856 | 0.839 |
|  | CONPOSITION | 0.762 | 0.743 | 0.828 | 0.778 | 0.633 | 0.614 | 0.755 | 0.667 |
|  | MethyRNA | 0.764 | 0.784 | 0.835 | 0.794 | 0.650 | 0.678 | 0.756 | 0.695 |
|  | EIIP | 0.763 | 0.743 | 0.829 | 0.778 | 0.632 | 0.614 | 0.755 | 0.667 |
|  | PseKNC | 0.732 | 0.708 | 0.825 | 0.755 | 0.600 | 0.582 | 0.706 | 0.630 |
|  | AutoCo | 0.665 | 0.677 | 0.712 | 0.684 | 0.538 | 0.546 | 0.593 | 0.559 |
|  | PSNP | 0.808 | 0.812 | 0.829 | 0.816 | 0.733 | 0.764 | 0.764 | 0.754 |
|  | onehot | 0.765 | 0.785 | 0.836 | 0.795 | 0.652 | 0.679 | 0.756 | 0.696 |

**Table S6 Performance of YTHDF3 target prediction using cross-condition test**

|  | Encoding Method | Full transcript model | | | | Mature mRNA model | | | |
| --- | --- | --- | --- | --- | --- | --- | --- | --- | --- |
|  |  | D13 | D14 | D15 | Average | D13 | D14 | D15 | Average |
| Cross  Validation | m6Areader | 0.978 | 0.978 | 0.978 | 0.978 | 0.830 | 0.830 | 0.830 | 0.830 |
|  | CONPOSITION | 0.822 | 0.842 | 0.834 | 0.832 | 0.749 | 0.815 | 0.639 | 0.734 |
|  | MethyRNA | 0.816 | 0.795 | 0.823 | 0.811 | 0.745 | 0.760 | 0.763 | 0.756 |
|  | EIIP | 0.822 | 0.842 | 0.834 | 0.832 | 0.749 | 0.815 | 0.611 | 0.725 |
|  | PseKNC | 0.811 | 0.833 | 0.811 | 0.819 | 0.742 | 0.807 | 0.615 | 0.721 |
|  | AutoCo | 0.792 | 0.810 | 0.794 | 0.799 | 0.721 | 0.778 | 0.609 | 0.703 |
|  | PSNP | 0.835 | 0.864 | 0.838 | 0.846 | 0.747 | 0.861 | 0.794 | 0.800 |
|  | onehot | 0.819 | 0.796 | 0.825 | 0.814 | 0.747 | 0.764 | 0.766 | 0.759 |
| Cross-condition Test | m6Areader | 0.987 | 0.989 | 0.999 | 0.992 | 0.882 | 0.854 | 0.912 | 0.883 |
|  | CONPOSITION | 0.825 | 0.794 | 0.727 | 0.782 | 0.787 | 0.726 | 0.606 | 0.707 |
|  | MethyRNA | 0.805 | 0.797 | 0.758 | 0.787 | 0.763 | 0.727 | 0.710 | 0.733 |
|  | EIIP | 0.825 | 0.794 | 0.727 | 0.782 | 0.788 | 0.727 | 0.667 | 0.727 |
|  | PseKNC | 0.792 | 0.763 | 0.705 | 0.753 | 0.759 | 0.703 | 0.655 | 0.706 |
|  | AutoCo | 0.771 | 0.746 | 0.696 | 0.737 | 0.735 | 0.684 | 0.646 | 0.688 |
|  | PSNP | 0.870 | 0.839 | 0.974 | 0.894 | 0.806 | 0.794 | 0.973 | 0.858 |
|  | onehot | 0.811 | 0.799 | 0.764 | 0.791 | 0.769 | 0.730 | 0.771 | 0.757 |

**Table S7 Performance of reader-specific target prediction evaluated using mixed sites**

|  |  | Method | YTHDC1 | YTHDC2 | YTHDF1 | YTHDF2 | YTHDF3 | EIF3A | Average |
| --- | --- | --- | --- | --- | --- | --- | --- | --- | --- |
| Full transcript model | Cross  Validation | m6Areader | 0.974 | 0.946 | 0.984 | 0.983 | 0.978 | 0.995 | 0.977 |
|  |  | Composition | 0.758 | 0.845 | 0.776 | 0.817 | 0.829 | 0.854 | 0.813 |
|  |  | MethyRNA | 0.749 | 0.790 | 0.801 | 0.824 | 0.818 | 0.808 | 0.798 |
|  |  | EIIP | 0.757 | 0.843 | 0.775 | 0.816 | 0.829 | 0.855 | 0.813 |
|  |  | PseKNC | 0.714 | 0.793 | 0.738 | 0.600 | 0.812 | 0.850 | 0.751 |
|  |  | AutoCo | 0.595 | 0.680 | 0.677 | 0.702 | 0.795 | 0.833 | 0.714 |
|  |  | PSNP | 0.749 | 0.880 | 0.783 | 0.616 | 0.834 | 0.910 | 0.795 |
|  |  | onehot | 0.753 | 0.790 | 0.810 | 0.824 | 0.822 | 0.801 | 0.800 |
|  | Independent  Set of Sites | m6Areader | 0.977 | 0.960 | 0.986 | 0.984 | 0.986 | 0.995 | 0.981 |
|  |  | Composition | 0.757 | 0.834 | 0.849 | 0.819 | 0.829 | 0.859 | 0.825 |
|  |  | MethyRNA | 0.757 | 0.781 | 0.809 | 0.826 | 0.820 | 0.793 | 0.798 |
|  |  | EIIP | 0.761 | 0.858 | 0.720 | 0.819 | 0.830 | 0.860 | 0.808 |
|  |  | PseKNC | 0.717 | 0.797 | 0.747 | 0.786 | 0.824 | 0.856 | 0.788 |
|  |  | AutoCo | 0.642 | 0.709 | 0.688 | 0.703 | 0.807 | 0.844 | 0.732 |
|  |  | PSNP | 0.779 | 0.844 | 0.796 | 0.804 | 0.836 | 0.898 | 0.826 |
|  |  | onehot | 0.757 | 0.756 | 0.798 | 0.825 | 0.820 | 0.817 | 0.796 |
| Mature mRNA model | Cross  Validation | m6Areader | 0.804 | 0.987 | 0.861 | 0.852 | 0.830 | 0.959 | 0.882 |
|  |  | Composition | 0.587 | 0.712 | 0.672 | 0.723 | 0.707 | 0.781 | 0.697 |
|  |  | MethyRNA | 0.593 | 0.811 | 0.712 | 0.726 | 0.757 | 0.733 | 0.722 |
|  |  | EIIP | 0.585 | 0.708 | 0.669 | 0.723 | 0.719 | 0.787 | 0.699 |
|  |  | PseKNC | 0.603 | 0.618 | 0.624 | 0.680 | 0.717 | 0.784 | 0.671 |
|  |  | AutoCo | 0.527 | 0.500 | 0.555 | 0.574 | 0.699 | 0.770 | 0.604 |
|  |  | PSNP | 0.686 | 0.883 | 0.716 | 0.729 | 0.768 | 0.877 | 0.777 |
|  |  | onehot | 0.632 | 0.617 | 0.713 | 0.726 | 0.762 | 0.733 | 0.697 |
|  | Independent  Set of Sites | m6Areader | 0.839 | 0.982 | 0.863 | 0.856 | 0.851 | 0.964 | 0.893 |
|  |  | Composition | 0.645 | 0.742 | 0.675 | 0.727 | 0.773 | 0.784 | 0.724 |
|  |  | MethyRNA | 0.635 | 0.814 | 0.716 | 0.726 | 0.765 | 0.735 | 0.732 |
|  |  | EIIP | 0.649 | 0.791 | 0.673 | 0.729 | 0.776 | 0.767 | 0.731 |
|  |  | PseKNC | 0.602 | 0.639 | 0.629 | 0.682 | 0.768 | 0.768 | 0.681 |
|  |  | AutoCo | 0.529 | 0.521 | 0.564 | 0.583 | 0.748 | 0.748 | 0.616 |
|  |  | PSNP | 0.740 | 0.879 | 0.718 | 0.729 | 0.798 | 0.862 | 0.788 |
|  |  | onehot | 0.649 | 0.567 | 0.715 | 0.733 | 0.764 | 0.742 | 0.695 |

Note: For each m^6^A reader, the targets identified by different experiments were mixed. Then, 80% of these targets were considered as training data, on which 5-fold cross-validation was performed. The trained predictors were then validated on the remaining 20% data for independent tests.

**Table S8 Comparison and evaluation of SVM, RF, LR and XGBoost**

| Gene Name | Values | Mature mRNA model | | | |
| --- | --- | --- | --- | --- | --- |
|  |  | SVM | RF | LR | XGBoost |
| EIF3a | Cross Validation | 0.959 | 0.959 | 0.959 | 0.959 |
|  | Independent test | 0.959 | 0.965 | 0.952 | 0.963 |
|  | PRAUC | 0.959 | 0.831 | 0.900 | 0.963 |
|  | Accuracy | 0.908 | 0.912 | 0.895 | 0.912 |
|  | MCC | 0.816 | 0.825 | 0.791 | 0.826 |
| YTHDF3 | Cross Validation | 0.830 | 0.830 | 0.830 | 0.830 |
|  | Independent test | 0.854 | 0.863 | 0.844 | 0.864 |
|  | PRAUC | 0.864 | 0.868 | 0.854 | 0.876 |
|  | Accuracy | 0.779 | 0.786 | 0.770 | 0.782 |
|  | MCC | 0.558 | 0.574 | 0.540 | 0.565 |
| YTHDC1 | Cross Validation | 0.808 | 0.841 | 0.771 | 0.852 |
|  | Independent test | 0.834 | 0.808 | 0.766 | 0.814 |
|  | PRAUC | 0.849 | 0.805 | 0.777 | 0.835 |
|  | Accuracy | 0.685 | 0.647 | 0.575 | 0.659 |
|  | MCC | 0.580 | 0.621 | 0.689 | 0.609 |
| YTHDC2 | Cross Validation | 0.606 | 0.752 | 0.622 | 0.785 |
|  | Independent test | 0.659 | 0.649 | 0.679 | 0.655 |
|  | PRAUC | 0.615 | 0.597 | 0.669 | 0.601 |
|  | Accuracy | 0.535 | 0.611 | 0.531 | 0.557 |
|  | MCC | 0.776 | 0.826 | 0.774 | 0.788 |
| YTHDF1 | Cross Validation | 0.859 | 0.863 | 0.853 | 0.872 |
|  | Independent test | 0.864 | 0.857 | 0.854 | 0.871 |
|  | PRAUC | 0.836 | 0.836 | 0.826 | 0.864 |
|  | Accuracy | 0.791 | 0.788 | 0.785 | 0.797 |
|  | MCC | 0.584 | 0.578 | 0.570 | 0.595 |
| YTHDF2 | Cross Validation | 0.854 | 0.851 | 0.846 | 0.867 |
|  | Independent test | 0.849 | 0.834 | 0.843 | 0.866 |
|  | PRAUC | 0.818 | 0.819 | 0.815 | 0.856 |
|  | Accuracy | 0.778 | 0.763 | 0.770 | 0.781 |
|  | MCC | 0.557 | 0.527 | 0.540 | 0.562 |

| Gene Name | Values | Full transcript model | | | |
| --- | --- | --- | --- | --- | --- |
|  |  | SVM | RF | LR | XGBoost |
| EIF3a | Cross Validation | 0.995 | 0.995 | 0.995 | 0.995 |
|  | Independent test | 0.994 | 0.996 | 0.961 | 0.995 |
|  | PRAUC | 0.989 | 0.984 | 0.962 | 0.989 |
|  | Accuracy | 0.972 | 0.979 | 0.941 | 0.974 |
|  | MCC | 0.945 | 0.959 | 0.883 | 0.948 |
| YTHDF3 | Cross Validation | 0.978 | 0.978 | 0.978 | 0.978 |
|  | Independent test | 0.985 | 0.985 | 0.985 | 0.987 |
|  | PRAUC | 0.987 | 0.986 | 0.987 | 0.989 |
|  | Accuracy | 0.955 | 0.959 | 0.953 | 0.960 |
|  | MCC | 0.910 | 0.919 | 0.906 | 0.921 |
| YTHDC1 | Cross Validation | 0.974 | 0.983 | 0.984 | 0.984 |
|  | Independent test | 0.976 | 0.980 | 0.978 | 0.981 |
|  | PRAUC | 0.981 | 0.941 | 0.984 | 0.985 |
|  | Accuracy | 0.949 | 0.949 | 0.951 | 0.951 |
|  | MCC | 0.862 | 0.861 | 0.866 | 0.866 |
| YTHDC2 | Cross Validation | 0.956 | 0.983 | 0.954 | 0.956 |
|  | Independent test | 0.920 | 0.947 | 0.902 | 0.902 |
|  | PRAUC | 0.907 | 0.831 | 0.852 | 0.852 |
|  | Accuracy | 0.892 | 0.867 | 0.817 | 0.817 |
|  | MCC | 0.733 | 0.686 | 0.597 | 0.597 |
| YTHDF1 | Cross Validation | 0.985 | 0.988 | 0.986 | 0.989 |
|  | Independent test | 0.986 | 0.987 | 0.986 | 0.988 |
|  | PRAUC | 0.978 | 0.944 | 0.973 | 0.984 |
|  | Accuracy | 0.960 | 0.965 | 0.959 | 0.964 |
|  | MCC | 0.921 | 0.930 | 0.919 | 0.929 |
| YTHDF2 | Cross Validation | 0.982 | 0.985 | 0.982 | 0.987 |
|  | Independent test | 0.983 | 0.987 | 0.983 | 0.987 |
|  | PRAUC | 0.975 | 0.956 | 0.974 | 0.982 |
|  | Accuracy | 0.954 | 0.956 | 0.952 | 0.955 |
|  | MCC | 0.909 | 0.913 | 0.905 | 0.912 |
