# Supplementary material for: m6A Reader: Epitranscriptome Target Prediction and Functional Characterization of N6-Methyladenosine (m6A) Readers
Source: Front Cell Dev Biol. 2020 Aug 11;8:741. doi: 10.3389/fcell.2020.00741 (PMC7431669; doi:10.3389/fcell.2020.00741)
Supplement: Supplementary file 3 [file Image_1.pdf]

Figure S1

A Human mature mRNA model of YTHDF1

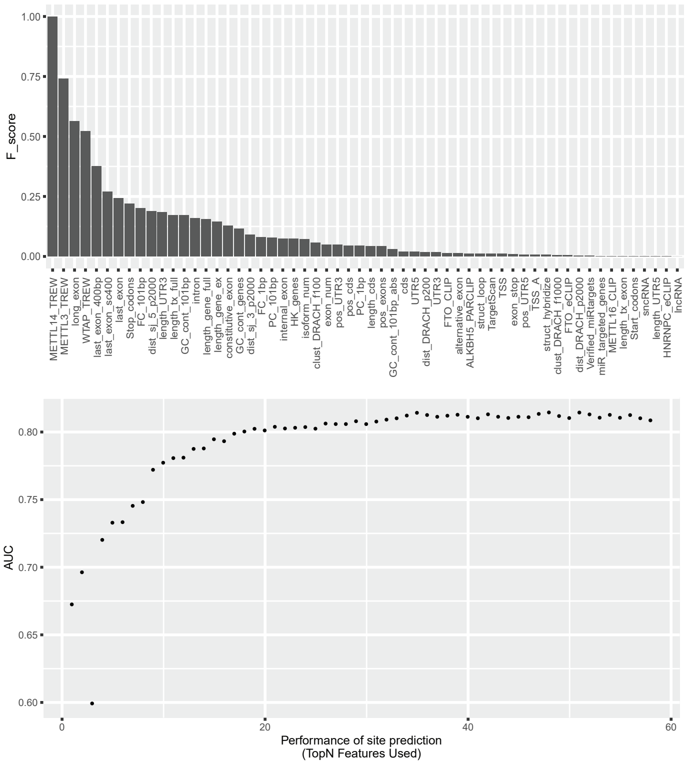

B Human full-transcript model of YTHDF1

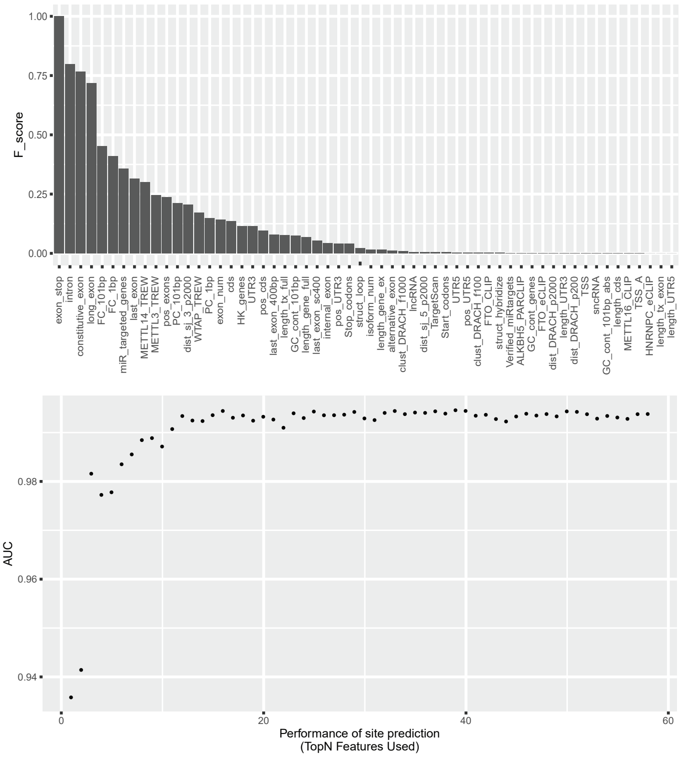

Figure S2

A Human mature mRNA model of YTHDF2

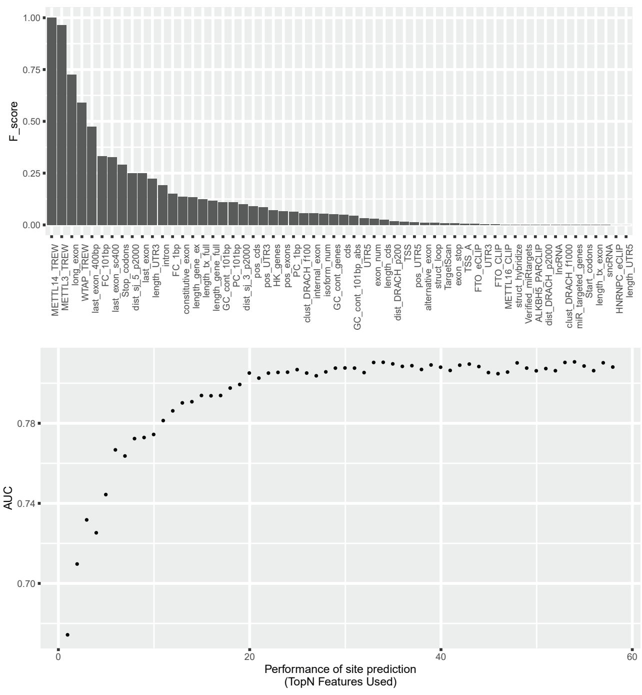

B Human full-transcript model of YTHDF2

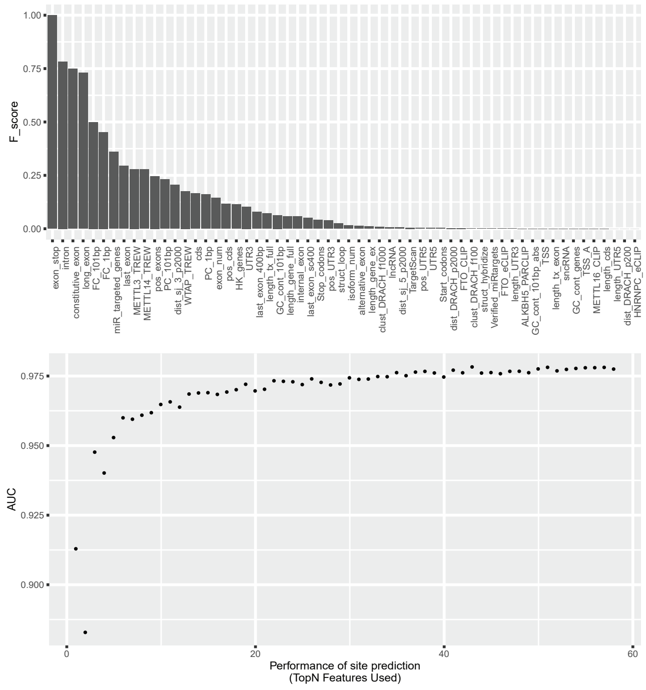

Figure S3

A Human mature mRNA model of YTHDF3

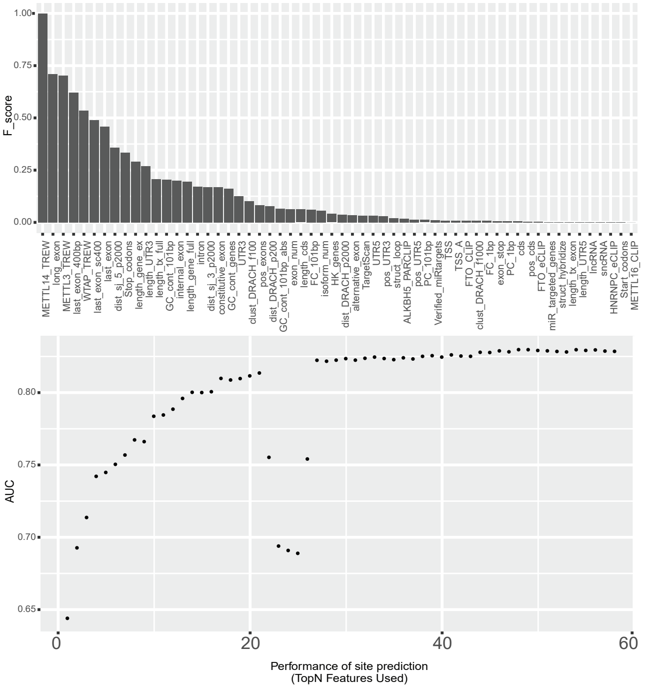

B Human full-transcript model of YTHDF3

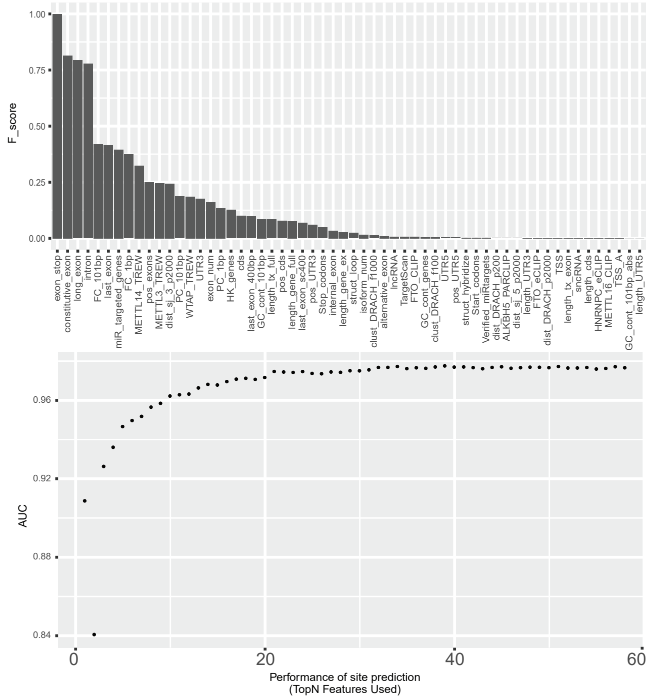

Figure S4

A Human mature mRNA mode of YTHDC1

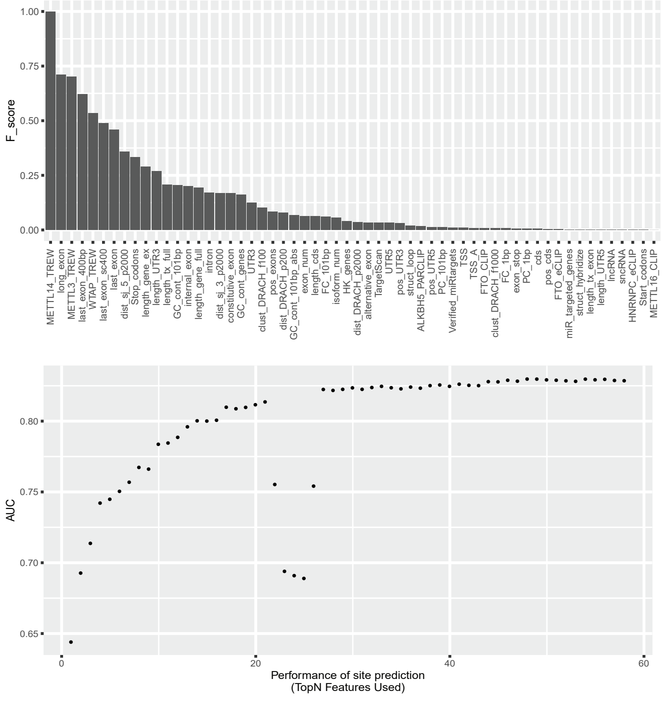

B Human full-transcript model of YTHDC1

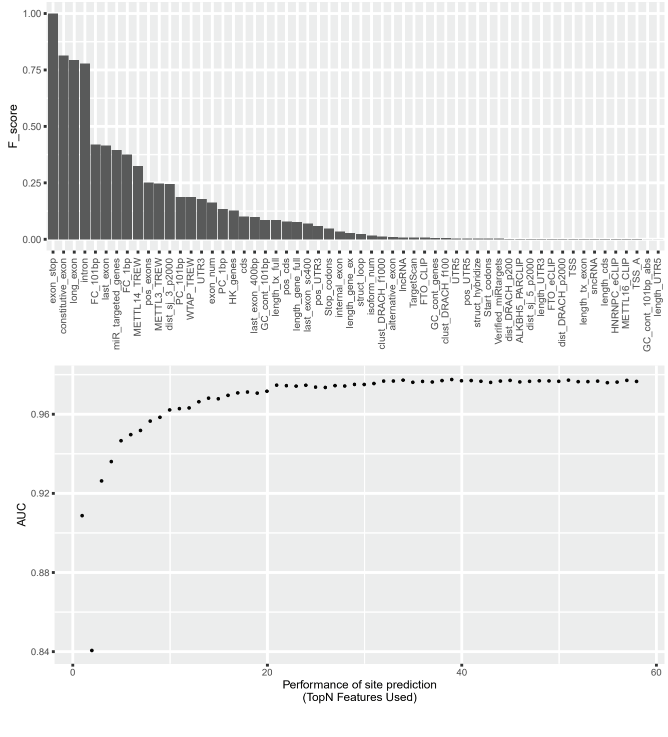

Figure S5

A Human mature mRNA mode of YTHDC2

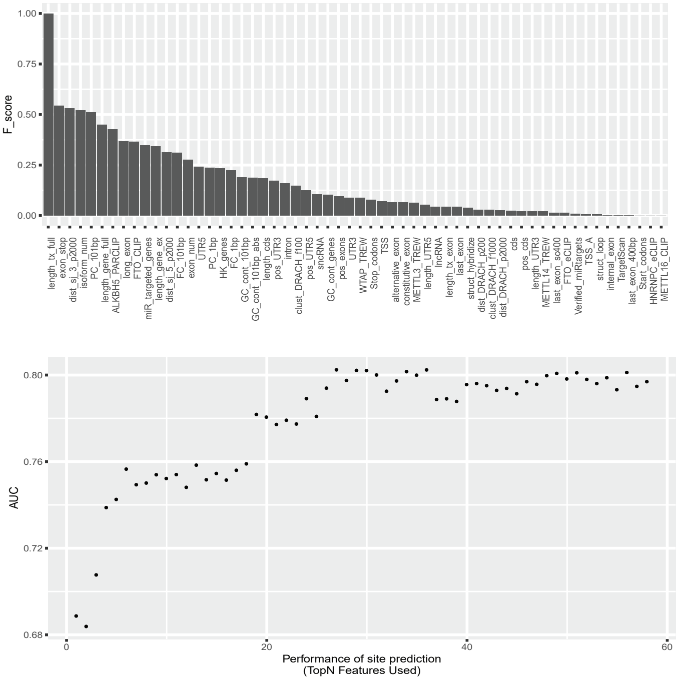

B Human full-transcript model of YTHDC2

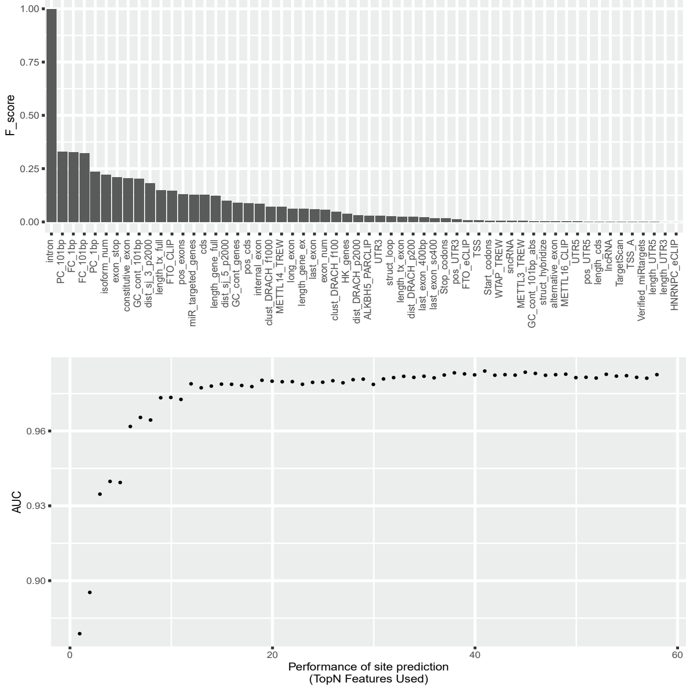

Figure S6

A Human mature mRNA mode of EIF3A

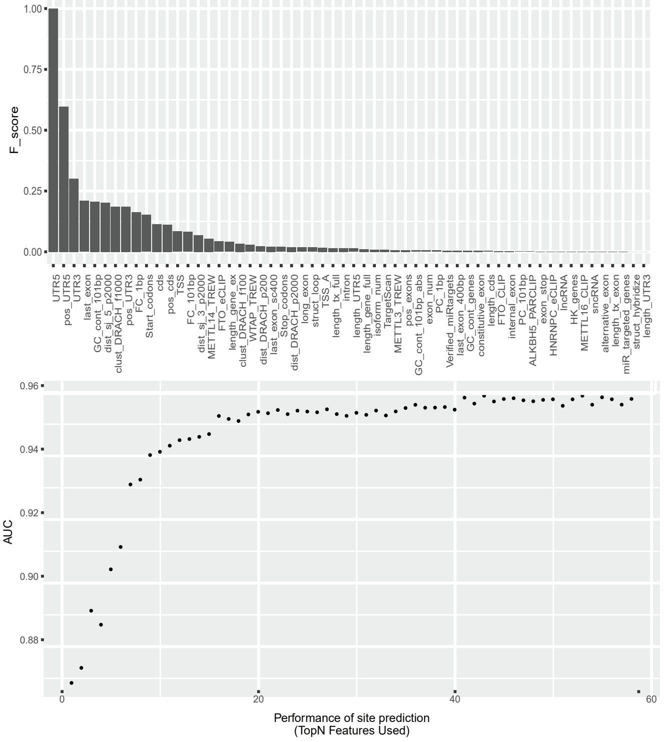

B Human full-transcript model of EIF3A

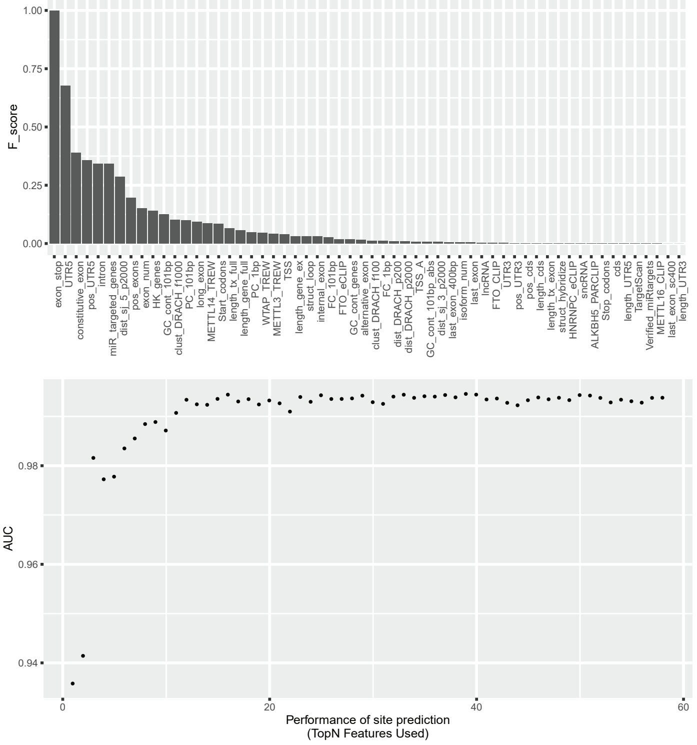

Figure S7.

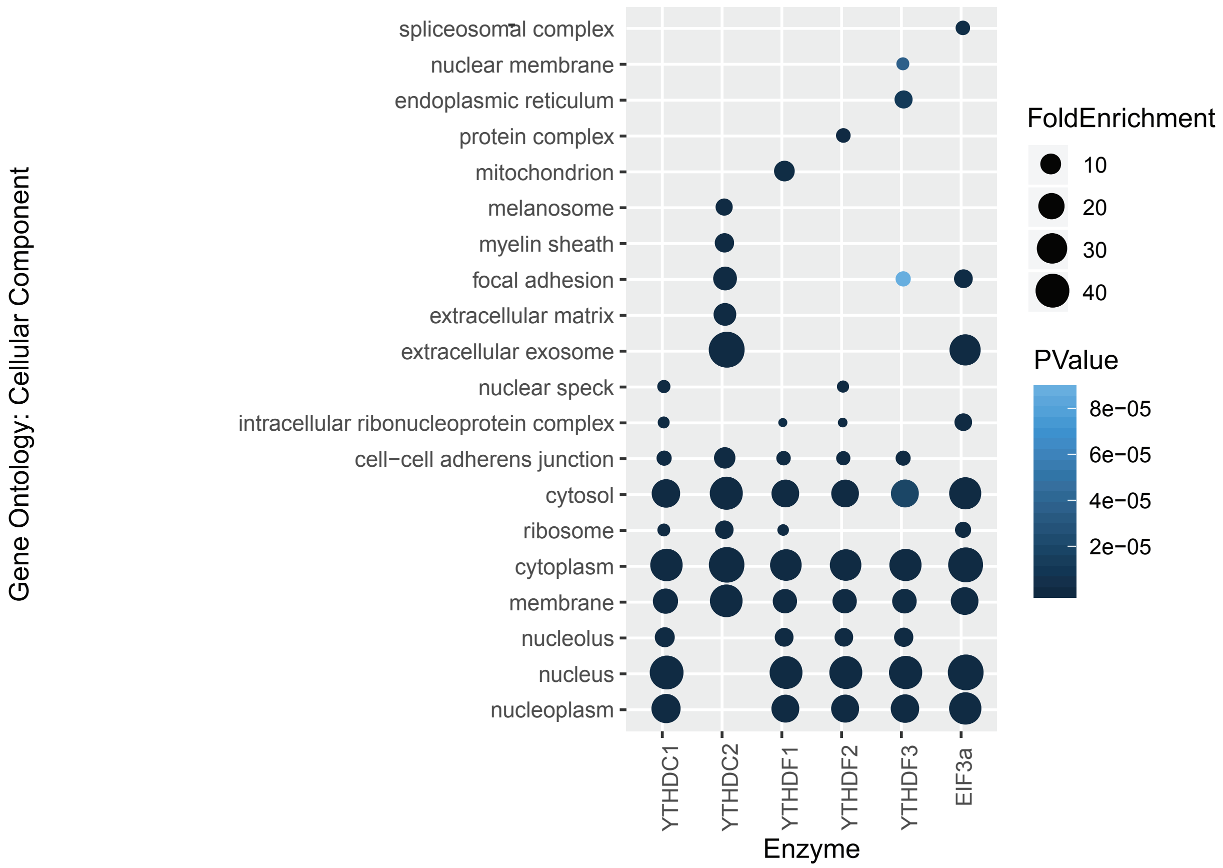
Gene Ontology: Cellular Component
spliceosomal complex
nuclear membrane
endoplasmic reticulum
protein complex
mitochondrion
melanosome
myelin sheath
focal adhesion
extracellular matrix
extracellular exosome
nuclear speck
intracellular ribonucleoprotein complex
cell−cell adherens junction
cytosol
ribosome
cytoplasm
membrane
nucleolus
nucleus
nucleoplasm
YTHDC1
YTHDC2
YTHDF1
YTHDF2
YTHDF3
EIF3a
Enzyme
FoldEnrichment
10
20
30
40
PValue
8e−05
6e−05
4e−05
2e−05

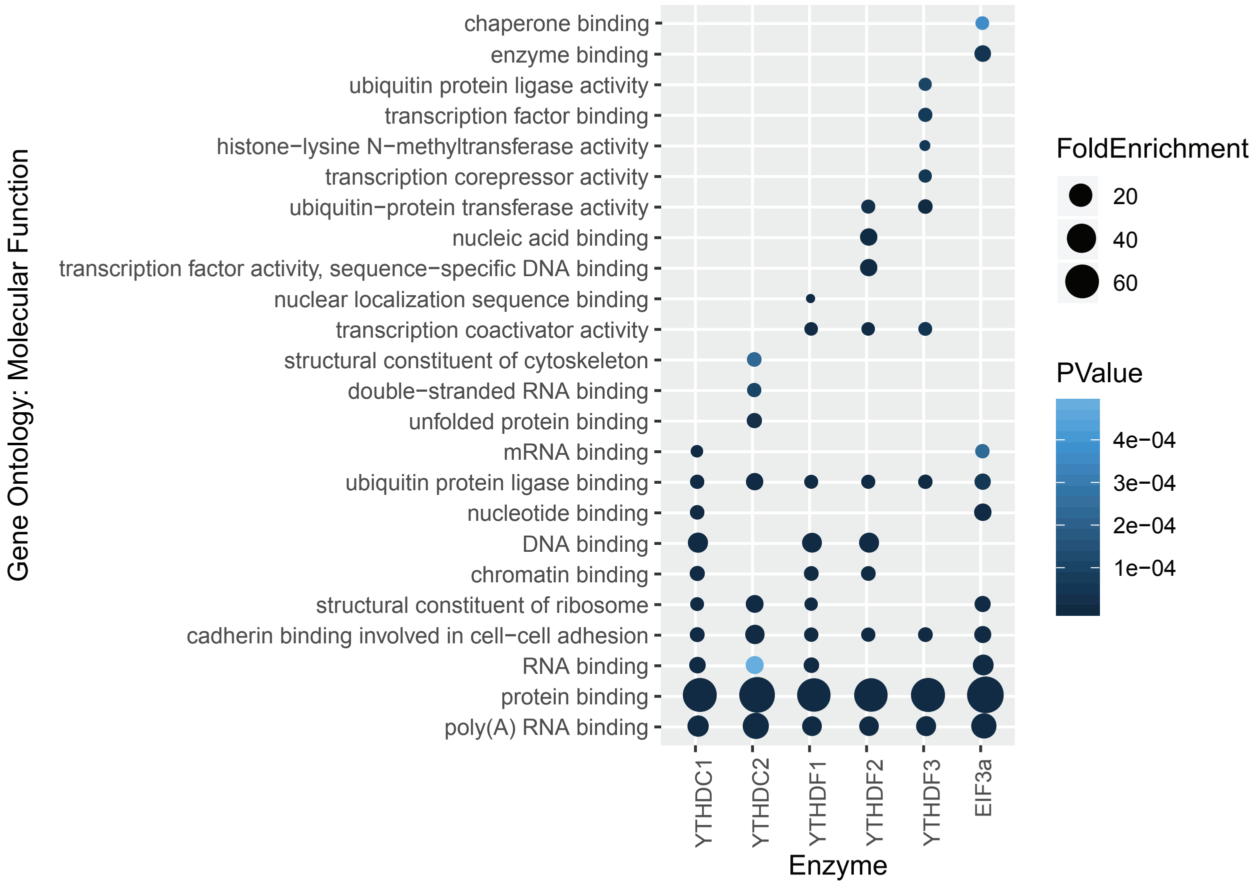
Gene Ontology: Molecular Function
chaperone binding
enzyme binding
ubiquitin protein ligase activity
transcription factor binding
histone−lysine N−methyltransferase activity
transcription corepressor activity
ubiquitin−protein transferase activity
nucleic acid binding
transcription factor activity, sequence−specific DNA binding
nuclear localization sequence binding
transcription coactivator activity
structural constituent of cytoskeleton
double−stranded RNA binding
unfolded protein binding
mRNA binding
ubiquitin protein ligase binding
nucleotide binding
DNA binding
chromatin binding
structural constituent of ribosome
cadherin binding involved in cell−cell adhesion
RNA binding
protein binding
poly(A) RNA binding
YTHDC1
YTHDC2
YTHDF1
YTHDF2
YTHDF3
EIF3a
Enzyme
FoldEnrichment
20
40
60
PValue
4e−04
3e−04
2e−04
1e−04
